# Alkaloid-Rich Crude Extracts, Fractions and Piperamide Alkaloids of *Piper guineense* Possess Promising Antibacterial Effects

**DOI:** 10.3390/antibiotics7040098

**Published:** 2018-11-09

**Authors:** Eunice Ego Mgbeahuruike, Pia Fyhrquist, Heikki Vuorela, Riitta Julkunen-Tiitto, Yvonne Holm

**Affiliations:** 1 Division of Pharmaceutical Biosciences, Faculty of Pharmacy, University of Helsinki, P.O. Box 56, FI-00014 Helsinki, Finland; Pia.fyhrquist@helsinki.fi (P.F.); Heikki.vuorela@helsinki.fi (H.V.); Yvonne.holm@helsinki.fi (Y.H.); 2 Natural Product Research Laboratory, Department of Environmental and Biological Sciences, University of Eastern Finland, 80101 Joensuu, Finland; Riitta.julkunen-tiitto@uef.fi

**Keywords:** Piperamides, antibacterial, *Piper guineense*, piperine, piperlongumine

## Abstract

*Piper guineense* is a food and medicinal plant commonly used to treat infectious diseases in West-African traditional medicine. In a bid to identify new antibacterial compounds due to bacterial resistance to antibiotics, twelve extracts of *P. guineense* fruits and leaves, obtained by sequential extraction, as well as the piperine and piperlongumine commercial compounds were evaluated for antibacterial activity against human pathogenic bacteria. HPLC-DAD and UHPLC/Q-TOF MS analysis were conducted to characterize and identify the compounds present in the extracts with promising antibacterial activity. The extracts, with the exception of the hot water decoctions and macerations, contained piperamide alkaloids as their main constituents. Piperine, dihydropiperine, piperylin, dihydropiperylin or piperlonguminine, dihydropiperlonguminine, wisanine, dihydrowisanine and derivatives of piperine and piperidine were identified in a hexane extract of the leaf. In addition, some new piperamide alkaloids were identified, such as a piperine and a piperidine alkaloid derivative and two unknown piperamide alkaloids. To the best of our knowledge, there are no piperamides reported in the literature with similar UVλ absorption maxima and masses. A piperamide alkaloid-rich hexane leaf extract recorded the lowest MIC of 19 µg/mL against *Sarcina* sp. and gave promising growth inhibitory effects against *S. aureus* and *E. aerogenes* as well, inhibiting the growth of both bacteria with a MIC of 78 µg/mL. Moreover, this is the first report of the antibacterial activity of *P. guineense* extracts against *Sarcina* sp. and *E. aerogenes*. Marked growth inhibition was also obtained for chloroform extracts of the leaves and fruits against *P. aeruginosa* with a MIC value of 78 µg/mL. Piperine and piperlongumine were active against *E. aerogenes*, *S. aureus*, *E. coli*, *S. enterica*, *P. mirabilis* and *B. cereus* with MIC values ranging from 39–1250 µg/mL. Notably, the water extracts, which were almost devoid of piperamide alkaloids, were not active against the bacterial strains. Our results demonstrate that *P. guineense* contains antibacterial alkaloids that could be relevant for the discovery of new natural antibiotics.

## 1. Introduction

Bacterial infections, resulting from bacterial contamination of food and water as well as from wound and post-operative infections are currently a menace in Sub-Saharan Africa [1]. The continuous increase in microbial resistance due to the gross misuse of antibiotics has led to a decline in the effectivity of antibiotic treatments. Consequently, there is a constant need for new antibacterial drugs with good efficacy, few and mild adverse effects and low cost. The increasing microbial resistance to antibiotics has led to the search for new antibacterial agents from medicinal plants [2] and medicinal plants such as *Piper guineense* and *Xylopia aethiopica*, which are used in the treatment of bacterial infections in West African traditional medicine, could be good sources for these compounds. Based on numerous pharmacological studies, there is growing interest in medicinal plants as sources of antibacterial agents and adjuvants [3,4]. It has been established that plant-derived compounds and plant extracts are potential sources for new antibacterial drugs against multi-drug-resistant (MDR) pathogens [3,5]. In many African countries, bacterial infections are among the main causes of diseases that affect the population and they often lead to life-threatening complications and can frequently result in death. Due to the prevalence of these bacterial infections and lack of adequate modern health facilities, low income earners in remote communities often practice self-medication, using herbal formulations for the treatment of bacterial infections. Moreover, a greater number of them seek medications from traditional medical practitioners (TMP), due to the high cost of treatment associated with the few modern health facilities [6]. The availability and accessibility of numerous medicinal plants in Africa has led to the use of plant extracts as herbal remedies for infectious diseases.

*Piper guineense* Schumach & Thonn, also known as African guinea pepper or African black pepper, is a medicinal plant that has a wide application in traditional medicine and is therefore of great ethnobotanical interest [7,8,9]. It is a perennial climbing vine reaching heights of 20 m. African black pepper has a pungent aroma and the fruits, leaves and roots are used for the preparation of herbal remedies for the treatment of infectious diseases [10,11]. In recent studies, *P. guineense* has been shown to be one of the most valuable spices with numerous health benefits [12]. In an ethnopharmacological survey conducted by Ajibesin et al. [13], *P. guineense* was found to be one of the major medicinal plants used for the treatment of various sexually transmitted diseases. Phytochemical studies have revealed that alkaloids, some of which could be novel drug compounds or scaffolds, are the main antimicrobial compounds present in *P. guineense* [14,15,16,17].

The aim of this study was to screen extracts of various polarities of *P. guineense* fruits and leaves and piperine and piperlongumine commercial compounds for antibacterial activity against significant human pathogenic bacteria including five Gram-negative and three Gram-positive bacteria. An agar well diffusion and a microdilution method were used for the screenings [18]. Moreover, selected extracts with promising antibacterial activities were investigated for their phytochemical composition using HPLC-DAD and UHPLC/Q-TOF MS in search of antibacterial extracts and compounds. Although *P. guineense* crude extracts have been previously reported to exhibit antibacterial properties [19,20,21,22,23], to the best of our knowledge, this is the first report on the antibacterial effects of *P. guineense* extracts of various polarities obtained by sequential extraction, supporting its ethnopharmacological uses. The present study is also the first report on the antibacterial evaluation of *P. guineense* extracts against *Enterobacter aerogenes* and *Sarcina* sp. Alsoverylittle is known about the antibacterial activity of the commercial piperamide compounds piperlongumine and piperine against these pathogenic bacterial strains. Since piperine and its related derivatives and synthetic piperine analogues are currently paving way as therapeutic agents for multiple human infectious diseases [24,25], there is a need to screen related piperamides against pathogenic multidrug-resistant bacteria such as *Pseudomonas aeruginosa*, *Escherichia coli* and *Staphylococcus aureus*.

## 2. Results

### 2.1. Antibacterial Activity

The results for the antibacterial activity of *P. guineense* extracts of various polarities are shown in Table 1 and Table 2. The bacteria used for the screenings were model human pathogenic bacteria that are of clinical importance. 

#### 2.1.1. Antibacterial Activity of *P. guineense* Extracts against *Pseudomonas aeruginosa*

**Figure 1:**
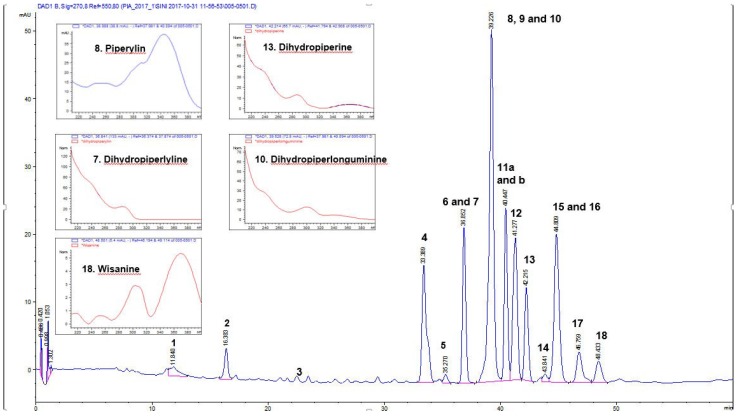
HPLC profile and identified piperamide alkaloids in an antibacterial hexane leaf extract of *P. guineense* at 270 nm. The bioactive compounds identified in this extract are also shown in Table 4. UVλ absorption maxima of some of the compounds are indicated.

Themethanol and chloroform extracts of the leaves and fruits of *P guineense* were effective against *Pseudomonas aeruginosa* with inhibition zones ranging from 15.3–21.3 mm (Table 1). In agreement with our results for the fruits, Eruteya et al. [26], reported that a methanol extract of *P. guineense* fruits gave the best growth inhibitory activity against *P. aeruginosa*. However, in our study, a chloroform leaf extract gave the largest inhibition zone of 21.3 mm, whereas a methanol leaf extract gave a significantly smaller IZ of 15.3 mm, which could be due to the higher concentrations of other piperamide alkaloids relative to piperine in the chloroform extract compared to the methanol extract of the leaves. Moreover, the minimum inhibitory concentrations (MIC) of the chloroform extracts were 78 µg/mL for both fruits and leaf, while the MIC of the methanol extracts were 1250 µg/mL for both the fruits and leaves and these results correlated well with the agar diffusion results for these extracts (Table 3). Our result demonstrates that chloroform extracts of the leaves of *P. guineense* containa high variety and concentration of piperamide alkaloids and other non-polar compounds that may be explored as antibacterial agents, alone and in combinations with antibiotics, for multidrug-resistant bacteria. In addition to the extracts, we studied the antibacterial effects of some piperamide alkaloids, such as piperine, which we have found to be present in high concentrations in *P. guineense* leaves (Figure 1) and piperlongumine, which is known in many species of *Piper*, although we did not find it in the leaves of *P. guineense*. Piperine gave moderate activity against *P. aeruginosa* with an inhibition zone of 13.3 mm and MIC of 156 µg/mL and thus, may not be the only bioactive compound responsible for the good growth inhibitory effects of the leaf chloroform extract of *P. guineense*. Thus, some other piperamide alkaloids in the leaf extract of *P. guineense* are suggested to be even more effective and should be tested alone for their growth inhibitory effects against *P. aeruginosa*. Piperlongumine was more effective against *P. aeruginosa* in terms of the sizes of its inhibition zones of (15.7 mm) but gave a higher MIC value (312 µg/mL) than piperine. However, it is interesting to note that the hexane and water extracts of both leaves and fruits of *P. guineense* were not active against *Pseudomonas aeruginosa.* This result might be explained by the absence of piperamide alkaloids in the water extracts. Moreover, the inactivity of the hexane extracts could possibly be reflected to their smaller quantity of piperamides when compared to the chloroform extracts. Our results demonstrate that *P. guineense* could be a source of new antibacterial agents or adjuvants that could be relevant for the treatment of infections associated with *Pseudomonas aeruginosa*.

#### 2.1.2. Antibacterial Activity of *P. guineense* Extracts against Bacillus Cereus

Extracts from *P. guineense* were susceptible to *B. cereus* with inhibition zones ranging from 15.3–29.3 mm (Table 2). The largest zones of inhibition (29.3 mm and 28.2 mm) were observed with the methanol and ethanol extracts of the leaves and this result correlated well with the MIC values for these extracts, which were found to be 78 µg/mL and 39 µg/mL, respectively. The inhibition zones for the methanol and ethanol extracts of the fruits were 23.7 mm and 24.5 mm, respectively and the MIC values of these extracts were 78 µg/mL and 39 µg/mL. Based on the result obtained with this bacterium, it is interesting to note that the leaf extracts were more active than the fruit extracts. It could be possible that the leaf contains more antibacterial compounds that could inhibit the growth of this particular bacterium. Moreover, we found that the hexane and chloroform extracts were active against *B. cereus* but in contrast the water extracts were not active. This result demonstrates that the piperamide alkaloids present both in the methanol and ethanol extracts, as well as in the hexane extract of the leaf (Figure 1) is important for the growth inhibitory effects of these extracts against *B. cereus*. Thus, the inactivity of the water extract could be related to the absence of piperamide alkaloids in this extract. 

Our results could justify the use of *P. guineense* ethanol extracts asherbal remedy for the treatment of diarrhoea related to foodborne diseases.

#### 2.1.3. Antibacterial Activity of *P. guineense* Extracts against Staphylococcus Aureus

**Figure 2:**
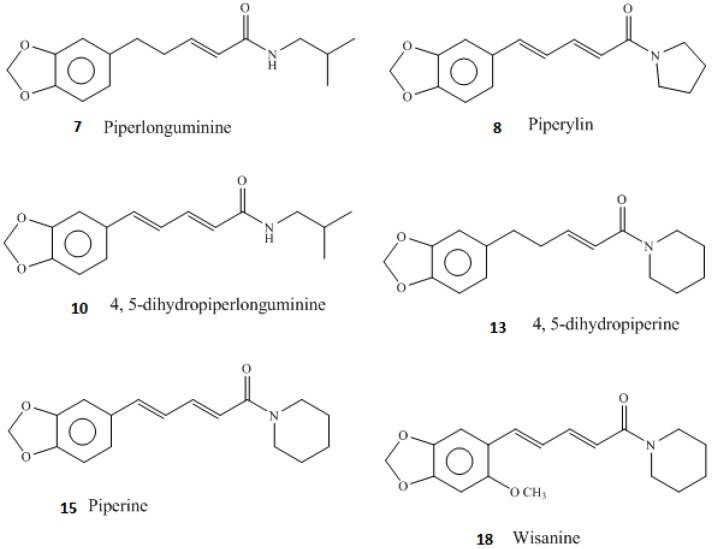
Chemical structures of some of the piperamide compounds found in this investigation from the hexane leaf extract of *Piper guineense*. The compounds are numbered from Table 4.

*P. guineense* extracts and pure compounds were active against *S. aureus* with inhibition zones ranging from 14.7–22.67 mm. In this study, the largest inhibition zone of 22.67 mm was observed with an ethanol extract of the fruits, followed by a leaf ethanol extract and chloroform and hexane extracts of the fruits (Table 2). For the ethanol extract of the fruits, our agar diffusion results correlated well with the MIC resultof 78 µg/mL (Table 3). However, this same MIC of 78 µg/mL could also be recorded for the ethanol extract of the leaves as well as the fruit hexane and chloroform extracts, even though these extracts produced smaller inhibition zones than the leaf ethanol extract. In contrast to the other extracts we found that the water extracts of both the leaves and fruits showed no inhibition. Thus, our result suggests that *P. guineense* should not be used as water extracts for the treatment of bacterial infections but rather the fruits or leaves should be soaked with alcohol. When compared to the extracts of *P. guineense*, we found that the piperamide alkaloids, present in the extracts, were not more growth inhibitory. Piperine was more active than piperlongumine, giving an inhibition zone of 18.3 mm and a MIC of 78 µg/mL, compared to 15.3 mm and 156 µg/mL for piperlongumine. This result indicates that piperine is one of the piperamide alkaloids in the ethanol, methanol, chloroform and hexane extracts of *P. guineense* which could be growth inhibitory against *S. aureus*. Our phytochemical profiling of the extracts using HPLC-DAD and UHPLC/QTOF-MS shows that *P. guineense* leaves contain a high variety of piperamide alkaloid derivatives which could all be critical for the antibacterial effects of these extracts (Table 4, Figure 2). Our result correlates with the previous report that piperlongumine has moderate activity against *S. aureus* [27]. Moreover, in agreement with our results, previous reports have also shown that the water extracts of *P. guineense* are usually less effective when compared to the methanol, ethanol and hexane extracts [23]. Our results on the antibacterial activity of the extracts of *P. guineense* against *Staphylococcus aureus* justify the use of alcohol extracts and soups containing this plant for the treatment of common foodborne and other infections caused by this bacterium.

#### 2.1.4. Antibacterial Activity of *P. guineense* Extracts against *Sarcina* sp.

*Sarcina* sp. was the most sensitive bacterium to extracts of *P. guineense*. The methanol, ethanol, chloroform and hexane extracts of *P. guineense* showed promising growth inhibitory activity with MIC values between 19–39 µg/mL and inhibition zones ranging from 25.67–37.67 mm (Table 2). The largest inhibition zones were observed with hexane fruit and leaf extracts, showing remarkable zones of inhibition of 37.67 and 33.67 mm, respectively. Accordingly, the hexane leaf extract also gave a low MIC of 19 µg/mL and was thus as effective as piperine which gave an identical MIC value (Table 3). The antibacterial activity of the hexane leaf extract and the other effective extracts can be attributed to the occurrence of piperine and other piperamide alkaloids as the main constituents in these extracts (Table 4, Figure 1). To the best of our knowledge, this is the first report on the growth inhibitory effects of *P. guineense* extracts on *Sarcina* sp. *Sarcina* sp. is an anaerobic Gram-positive bacterium belonging to the family Clostridiaceae [28]. *Sarcina* has mainly been characterized as the causative agent of abomasal bloat leading to the death of livestock [29]. There is still a debate whether *Sarcina* is pathogenic to humans, although a number of cases of human disease, including gastric perforation, emphysematous gastritis and gastric ulcer have been associated with *Sarcina* [28,29,30]. Our result demonstrates that the hexane extracts of both *P. guineense* fruits and leaves contain antibacterial piperamide alkaloids, which could be used as therapeutic agents in the treatment of *Sarcina* infections. The findings justify the traditional use of *P. guineense* alcoholextracts for the treatment of stomach related problems, including gastric ulcers. 

#### 2.1.5. Antibacterial Activity of *P. guineense* Extracts against Proteus Mirabilis

*P. mirabilis* was found to be rather resistant against most of the extracts of *P. guineense*. In the primary screening, using agar diffusion, we found that the ethanol and methanol extracts were more active than the chloroform fruit and leaf extracts (Table 1). However, when the microdilution method was used for estimating the MIC values, we found large variations in activity between the extracts demonstrating MIC values between 78 and 2500 µg/mL. From these results the ethanol extracts of the fruits and leaves stood out with very low MIC values of 78 µg/mL, thus demonstrating that ethanol could be a good solvent for extracting compounds active against *P. mirabilis* (Table 3). Contrary to our results, Okeke et al. [31], reported that *P. guineense* fruit extract is not active against *Proteus mirabilis.* However, they found activity against *Proteus vulgaris.* We found that piperine showed significant activity against *P. mirabilis* with an inhibition zone of 16.0 mm and a MIC value of 78 µg/mL and a MBC value of 156 µg/mL. Thus, piperine must be responsible for a part of the good antibacterial activity of the ethanol extracts of the fruits and leaves against *P. mirabilis*, since piperine was observed to be present in both extracts according to our preliminary HPLC-DAD data (Figure 1). In contrast to piperine, we found that piperlongumine was not active against *P. mirabilis*. Thus, piperamide alkaloids vary in respect to their antimicrobial activities, even though they would be closely related to their molecular structures. Our results now demonstrate that especially ethanol extracts of *P. guineense* fruits and leaves could be used for the treatment of urinary tract infections with *Proteus mirabilis* as the main causative agent.

#### 2.1.6. Antibacterial Activity of *P. guineense* Extracts against *Enterobacter aerogenes*

We found that a methanol extract of the fruit gave the largest zone of inhibition (49.7 mm) of all tested extracts against *E. aerogenes* and the effect was comparable to tetracycline (45.3 mm) (Table 1). Moreover, our MIC value for this extract, 39 µg/mL, correlated well with the large inhibition zone (Table 3). The methanol leaf extract showed an inhibition zone of 29.3 mm and a large inhibition zone of 34.7 mm was also observed for the hexane-fruit extract, both extracts showing MIC values of 39 µg/mL. The hexane leaf extract and ethanol fruit and leaf extracts showed promising inhibitory activity with MIC values of 39 µg/mL and 78 µg/mL respectively. However, the size of the inhibition zone of the hexane leaf extract, 17.8 mm, indicating just slight activity, did not correlate well with the low MIC of 78 µg/mL of this extract (Table 1 and Table 3). This result might be due to the hydrophobic piperamide alkaloids present in the leaf hexane extract not diffusing well into the agar and thus producing small zones of inhibition. The chloroform fruit-extract was moderately active with a MIC value of 312 µg/mL. To the best of our knowledge, this is the first report on the growth inhibitory activity of the extracts of *P. guineense* against *E. aerogenes*. Moreover, we found that the piperamidespiperine and piperlongumine were growth inhibitory against *E. Aerogenes* with inhibition zones of 18.7 mm and 12.7 mm, respectively, MIC values of 39 µg/mL and 156 µg/mL, respectively and MBC values of 78 µg/mL and 312 µg/mL, respectively. Thus, piperine which we have found to occur in all extracts, except from water extracts, must be responsible for part of the activity of the methanol, ethanol and hexane extracts of the fruits and leaves against *Enterobacter aerogenes*. Notably, the water extracts lacking piperamide alkaloids, were devoid of activity. *Enterobacter aerogenes* is a Gram-negative pathogenic bacterium that is responsible for most hospital-acquired infections [32]. It causes urinary tract infections, lower respiratory tract infections, skin and soft tissue infections. 

#### 2.1.7. Antibacterial Activity of *P. guineense* Extracts against *Escherichia coli*

We recorded the lowest MIC of 156 µg/mL against *E. coli* for an ethanol extract of the leaves of *P. guineense*, which correlated well with an inhibition zone of 19.8 mm (Table 1 and Table 3). The methanol fruit and leaf extracts of *P. guineense* also gave large inhibition zones of 20.7 mm and 20.3 mm, respectively. The chloroform extracts of the fruits and leaves were also effective, both giving an inhibition zone of 20.3 mm. Oppositely to the other extracts, the hexane and water extracts of *P. guineense* were not active against *E. coli*. For the hexane extract, this result is contradictory since this extract resembles the methanol and ethanol extracts to its piperamide alkaloid content. Therefore, in the methanol and ethanol extracts of the leaves and fruits of *P. guineense* there must be some compounds responsible for the activities which are not present in the hexane extracts. The result further suggests that standardized *P. guineense* extracts could be used for possible alternative treatment against pathogenic multidrug-resistant *E. coli* strains. This present study suggests that *P. guineense* extracts could contain new antimicrobial agents for multidrug-resistant pathogens. Furthermore, we found that the two piperamide pure compounds, piperine and piperlongumine, were effective against *E. coli* with inhibition zones of 22.3 mm and 20.0 mm respectively and MIC values of 19 and 39 µg/mL, respectively. This result could indicate that the antibacterial activity of *P. guineense* methanol and ethanol extracts against *E. coli* is partly due to the mentioned piperamide alkaloids. Our results could justify the use of *P. guineense* ethanol extracts of the fruits and leaves for the treatment of diarrhoea in African traditional medicine. 

#### 2.1.8. Antibacterial Activity of *P. guineense* Extracts against *Salmonella enterica*

The ethanol fruit extract of *P. guineense* showed the largest zone of inhibition of 21.7 mm for *S. enterica*, and MIC and MBC values of 78 µg/mL and 156 µg/mL, respectively. The ethanol leaf extract also showed a clear inhibition zone of 16.2 mm and a MIC of 78 µg/mL (Table 1 and Table 3). These results are in accordance with a previous investigation which demonstrates that ethanol extracts of *P. guineense* are growth inhibitory against *S. enterica* serovar enteritis [33]. However, oppositely to our results, Temitope [33], also found that water extracts of *P. guineense* inhibit the growth of *S. enterica*. These differences might be due to Temitope [33] using a different serovar of *S. enterica*. The result specifically demonstrates that, the more polar ethanol extracts contain antibacterial compounds that effectively inhibited the growth of this bacterium. Moreover, we found that in addition to the ethanol extracts also chloroform, hexane and methanol extracts gave some growth inhibition against *S. enterica*, with inhibition zones up to 18.0 mm. Piperine and piperlongumine showed clear inhibition zones of 14.7 and 11.3 mm with MIC values of 78 µg/mL and 625 µg/mL.

### 2.2. UHPLC/QTOF-MS Results

HPLC-DAD and UHPLC/Q-TOF MS were used for phytochemical profiling of antibacterial extracts of *P. guineense* and for the identification of piperamide alkaloids in *Piper guineense*. Our HPLC-DAD data indicates that piperamide alkaloids are the predominant compounds in *P. guineense* hexane, methanol, ethanol and chloroform extracts (Figure 1) and these compounds are suggested to be responsible for the antibacterial effects we have observed for this plant in agreement with other authors [7,34]. For proper identification and characterization of the piperamide alkaloids, a hexane extract of the leaf was chosen. This is because the chromatographic peaks in the hexane extract were more clearly visible and better resolved when compared with the chloroform, methanol and ethanol extracts and moreover, the HPLC-DAD profile of the hexane extract was still found to be very similar to those of the other extracts. In addition, the hexane leaf extract was chosen for phytochemical analysis since we observed large inhibition zones for this extract against most of the bacterial strains, as well as the lowest MIC result of 19 µg/mL against *Sarcina* sp. Our UHPLC/QTOF-MS result of a leaf hexane extract of *P. guineense* is presented in Table 4. Altogether 18 compounds were identified, of which the majority were piperamide alkaloids. Among these compounds, the previously known piperamidealkaloids piperine, dihydropiperine, piperylin, dihydropiperylin or piperlonguminine, dihydro-piperlonguminine, wisanine, dihydrowisanine and various derivatives of piperine were identified. These derivatives of piperidinewere identified for the first time in this plant extract (compounds **9**, **10b**, **11** and **11b** in Table 4). The mass spectrometric data of these compounds were compared to the literature on piperamide alkaloids in *Piper* species [15,35,36]. 

In our UHPLC/QTOF-MS runs, positive ion mode was used to get the [M + Na]^+^ or [M + H]^+^ ions of the expected piperamide alkaloids. Papers on piperamide alkaloids which have been found from *Piper* spp. were used as references [15,16,35]. Among the piperamide alkaloids we have found in the leaves of *P. guineense*, the following could be identified and some of them in high quantities. The exact calculated mass of dihydropiperylin or piperlonguminine (**7**), Rt UHPLC12.967 min, was deduced to be 273.13648 according to the molecular formula of C_16_H_19_NO_3_. The [M + Na]^+^ ion for dihydropiperylin was 296.1250 and that of piperylin (**8**) at tR UHPLC 13.567 min was found to be 294.110. Both compounds were present also in the fruit extracts of *P. guineense*. Moreover, piperine (**15**) at tR 44.809 min (HPLC-DAD), showing a [M + Na]^+^ ion at *m*/*z* 308.1245 and dihydropiperine (**13**) at tR 42.215 min (HPLC-DAD), with a [M + Na]^+^ ion at *m*/*z* 310.1417 were present in the leaf extract. To confirm the identity of compound **15** to be piperine, we compared the UVλ max-spectrum and the retention time of this compound to a commercial piperine reference compound and the data was found to match. Piperine has been previously reported to be present in in *P. guineense* extracts of the fruits [16]. Wisanine (**18**) at tR 48.429 min with a [M + Na]^+^ ion at *m*/*z* 338.1371 and dihydrowisanine (**16**) at tR 44.848 min with a [M + Na]^+^ ion at *m*/*z* 340.1530 were also found to be present in the hexane leaf extract. Moreover, some derivatives of piperidine (compounds **9**, **10b**, **11** and **11b** in Table 4) were identified for the first time in this plant extract. Wisanine and dihydrowisanine were present also in the chloroform, ethanol, methanol and hexane extracts according to our HPLC-DAD results. The other alkaloids found in the extracts include: Dihydropiperlonguminine (**10**) (tR 39.226 min, [M + Na]^+^ ion at *m*/*z* 298.1337); a piperine derivative composed of piperine + 1 oxygen- one double bond (**10b**) (tR 39.249 min, [M + Na]^+^ ion at *m*/*z* 324.1211); an unknown piperamide alkaloid (**11**) (tR 40.447 min, [M + Na]^+^ ion at *m*/*z* 358.1528); and a dihydro derivative of piperidine (**9**) (tR 39,226 min, [M + Na]^+^ ion at *m*/*z* 326.1374) (Table 4).

## 3. Discussion

In this study, *Piper guineense* fruit and leaf extracts and piperamide alkaloid compounds have revealed potent antibacterial activity against Gram-positive and Gram-negative bacteria. *P. guineense* is used by traditional healers in West Africa for various medicinal purposes and often, as herbal remedies for the treatment of symptoms related to bacterial infections, such as diarrhoea, cough and rashes as well as for the treatment of infected wounds and thus these uses are now justified by our results.

In our study, we used *Pseudomonas aeruginosa* as a Gram-negative model bacterium, since it is capable of causing life-threatening nosocomial infections and many combination therapy resistant strains have been reported [37,38]. In recent times, because of the increase in microbial resistance, only few antibiotics are effective in the treatment of the multidrug-resistant (MDR) and extensively drug-resistant (XDR) lineages of *Pseudomonas aeruginosa* infections [39]. Our results reveal potent growth inhibitory effects of both leaf and fruit chloroform extracts of *P. guineense* against *P. aeruginosa*, both giving MIC values of 78 μg/mL against this bacterium. This result agrees with Eruteya et al. [26], who also reported that extracts of *P. guineense* fruits give good growth inhibitory activity against *P. aeruginosa*.

*Staphylococcus aureus* is becoming increasingly resistant to conventional drug therapies, with MRSA and VRSA strains which are not responding well to any drugs [5]. Previous research has shown that plant-derived compounds and plant extracts could be used as novel treatments and sources of new antibacterial compounds against multi-drug-resistant (MDR) pathogens, including methicillin-resistant *Staphylococcus aureus* (MRSA) [5]. Our results indicate that alcohol extracts as well as chloroform and n-hexane extracts from the fruits and leaves of *P. guineense* could be used as good sources for piperamide alkaloids which could be used for treatment of infections caused by *S. aureus*. Interestingly, we found that the extracts were as growth inhibitory as the pure compounds, piperine and piperlonguminine. This suggests that some of the other alkaloids in the extracts could be even more powerful and warrants for further isolation of piperamide alkaloids from *P. guineense* to test their effects against *S. aureus*.

*E. coli* is a Gram-negative bacterium that is capable of causing infectious diarrhoea in humans [40]. We found that methanol, ethanol and n-hexane extracts of *P. guineense* were active against this bacterium. Thus, we recommend that alcohol extracts of *P. guineense* leaves and fruits could be used for the treatment of *E. coli* infections. Our promising resultson the inhibitory capacity of especially ethanol extracts of the fruits of *P. guineense* against *E. coli* would prompt to study the effects of alcohol extracts also against strains of *E. coli*. Extracts of various polarities, such as methanol, ethanol, chloroform, hexane, cold water macerations and hot water decoctions of the fruits and leaves were screened for antibacterial activity.

We found that piperine and piperlongumine commercial compounds were active against most of the bacteria investigated in our study, with piperlongumine showing the lowest MIC value of 9.7 µg/mL against *Sarcina* sp. Piperine and piperlongumine showed significant activity against *E. coli* with MIC values of 19 µg/mL and 39 µg/mL, respectively. Piperine has been previously reported to give significant antibacterial activity against some bacterial strains [24,25]. Piperine isolated from the fruits of black pepper (*Piper nigrum*) was found to be effective against *Pseudomonas aeruginosa* and gave a MIC of 250 µg/mL against this bacterium [41] and this result is in accordance with our MIC of 156 µg/mL. At low concentrations of 0.1–10 µg/mL, piperine was found to reduce swarming and swimming motility of *E. coli* [42]. Moreover, piperine was found to be active against *S. aureus* with a MIC of 250 µg/mL and reduced the MIC of mupirocin fourfold and of ciprofloxacin eightfold [43], [44] and was found to inhibit efflux pumps in *S. aureus* [43].

In our screenings we found that the cold water macerations and hot water decoctions of the fruits and leaves did not show any inhibitory activity against most of the bacteria used for the study. The reason for water extracts lacking antibacterial activity could be attributed to the fact that the bioactive compounds present in the other extracts, which are mostly piperamide alkaloids, are not soluble in water and not even in boiling water according to our HPLC-DAD fingerprints of water extracts. However, in contradiction to our results, there are some reports on the antibacterial effects of water extracts of *Piperguineense* against *Salmonella enterica* [33]. The growth inhibitory activity of the water extract in this report might be attributed to the high concentrations used (20–60 mg/mL) and to some other compounds than piperamide alkaloids, such as phenolic acids. Thus, when *P. guineense* extracts are to be used for the treatment of bacterial infections in traditional medicine, our result now recommend that oily or ethanol preparations should be used. Our results on the chloroform extracts of *P. guineense* possessing the largest activity against *P. aeruginosa* differs from previous studies stating that methanol extracts of *P. guineense* are usually the most active ones against this bacterium [26].

From our UHPLC/QTOF-MS analysis conducted on the *P. guineense* extracts, it could be seen that the extracts were rich in alkaloids and thus their antibacterial activities could be mostly attributed to the presence of numerous piperamides (Table 4, Figure 1). The chemical structures of some of the piperamide compounds identified in this study are shown in Figure 2. Of the piperamides we have found in a hexane leaf extract of *P. guineense*, wisanine, dihydrowisanine, piperine, piperlongumine, 4,5-dihydropiperlonguminine and 4,5-dihydropiperylin have been earlier identified in *Piper guineense* fruits [15,16,35]. In addition to these piperamides we found some new structures, such as a piperine and a piperidine alkaloid derivative (compounds **9** and **10b** in Table 4) and two unknown piperamide alkaloids (compounds **11** and **11b** in Table 4). To the best of our knowledge to date there are no piperamides reported in the literature with the same UVλ maxima and masses.

## 4. Materials and Methods

### 4.1. Plant Material Collection

The fruits and leaves of *P. guineense* used in this study were collected from a rural village in Imo State, South Eastern Nigeria. The plant materials were authenticated at the Department of Crop Science of the Federal University of Technology, Owerri, Nigeria. Voucher specimens are deposited in the herbarium of the Department of Crop Science of the same university with the specimen number FUTO/SAAT/NS/005A for the fruit and FUTO/SAAT/NS/005B for the leaf. The reference flora used to identify the species is “Useful Plants of West Tropical Africa.” The species commonly occur in South Eastern Nigeria and the geographical coordinates of occurrence of the tested species is 5°45′00″ N, 8°30′00″ E.

### 4.2. Extraction

The air dried plant materials were milled with a grinder to obtain finely ground powdery samples. Sequential extraction was carried out using solvents of varying polarities, starting with the least polar solvent. First, 40 g of the plant material was extracted with 300 mL of hexane, followed by extraction with 300 mL of chloroform, then 300 mL of ethanol and the residue was finally extracted and washed with 300 mL of methanol. Seed and leaf plant materials were used for the extractions. For each of the solvents used, the extraction was conducted in duplicates and each extract was filtered using filter paper (Whatman GE Healthcare, Chicago, IL, USA) into a flask of known weight. The filtrate was evaporated using a Rotavapor (Heidolph VV2000) combined with a water bath not exceeding +40 °C, thereafter the extracts were lyophilized for two days to dry completely. The extraction yield for the extractsare as follows: hexane fruit extract (5.3%), hexane leaf extract (1.4%), chloroform fruit extract (8.9%), chloroform leaf extract (4.7%), ethanol fruit extract (8.5%), ethanol leaf extract (5.0%), methanol fruit extract (10.5%) and methanol leaf extract (6.0%). Macerations and hot water decoctions were also prepared from the plant samples since these preparations are used in traditional medicine. Macerations were prepared by weighing 10 g of the fruit and leaf plant materials into Erlenmeyer flasks. 100 mL of water was added and extraction was performed for 24 h using a magnetic stirrer. The mixture was centrifuged at 2000 rpm for 15 min (Eppendorf AG centrifuge 5810R, Germany). For the decoctions, 10 g of the plant material was boiled with 100 mL of water and allowed to cool. The mixture was centrifuged for 15 min at 2000 rpm (Eppendorf AG centrifuge 5810R, Hamburg, Germany). Both the macerations and decoctions were carefully filtered using filter paper (Schleicher & Schuell, ∅ = 150 mm, Hamburg, Germany) and freeze dried for two days in a lyophilizer. The extraction yields are as follows: decoction of the seed (2.8%), decoction of the leaf extract (2.4%), maceration of the seed (3.0%) and maceration of the leaf (2.8%). Prior to the agar diffusion test, the freeze dried extracts were reconstituted and re-dissolved in their corresponding solvents or in MeOH to a final concentration of 50 mg/mL for the antibacterial screening according to the method of Anyanwu and Nwosu [45] and Salih et al. [46].

### 4.3. Methods of Analytical Chemistry

#### 4.3.1. HPLC-UV/DAD Method 

The liquid chromatographic system consisted of aHPLC Waters 600 E pump and a controller coupled to a 991 photodiode array detector. Samples were injected using an autosampler controlled by Agilent Chemstation software (Waters Corp., Milford, TX, USA). A HypersilRp C18 column was used for the separations (length: 60 mm; internal diameter: 2 mm). 5–20 μL of the extracts (5–10 mg/mL in 100% MeOH) were injected. Gradient runs were performed using two solvent systems: A (aqueous 1.5% tetrahydrofuran + 0.25% orthophosphoric acid) and B (100% MeOH). Flow rate was 2 mL/min. UV fingerprint chromatograms were constructed at 220, 270, 280, 320 and 360 nm. UVλ absorption maxima spectra of the major compounds in the *P. guineense* extract were recorded between 200 and 400 nm using Agilent Chemstation software. The compounds in the extracts were identified by comparing their retention times and UVλ absorption maxima spectra with those of commercial reference standards (piperine and piperlongumine) and previous literatureon HPLC-DAD data for piperamide alkaloids was also used [15,16].

#### 4.3.2. UHPLC/Q-TOF MS Method

The masses of piperamide alkaloids in a leaf hexane extract were identified using UHPLC-DAD (Model 1200 Agilent Technologies)-JETSTREAM/QTOFMS (Model 6340 Agilent Technologies) equipped with a 2.1 × 60 mm, 1.7 μm C18 column (Agilent technologies) according to Taulavuori et al. [47]. The gradient range was from 0–50% of solvent A (aqueous 1.5% tetrahydrofuran + 0.25% acetic acid) and solvent B (100% methanol) and flow rate 0.4 mL/min. The qt of mass spectra were acquired at positive ion mode and mass rangefrom 100–2000 *m*/*z* was used. 

### 4.4. Bacterial Strains

The bacterial strains used in this investigation were obtained from the Division of Pharmaceutical Biosciences, Faculty of Pharmacy, University of Helsinki, Finland. In all, the growth inhibitory activity of the extracts were investigated using the Gram-positive strains *Sarcina* sp. FOMK (Division of Pharmaceutical Biosciences, University of Helsinki), *Staphylococcus aureus* ATCC 25923 and *Bacillus cereus* ATCC 10987 and the Gram-negative strains *Proteus mirabilis* 43071, *Enterobacter aerogenes* ATCC13048, *Escherichia coli* ATCC 25922, *Pseudomonas aeruginosa* ATCC 27853 and *Salmonella enterica* ATCC 43845).

#### 4.4.1. Antibiotics and Pure Compounds

Tetracycline hydrochloride (Sigma-Aldrich, St. Louis, MO, USA) and rifampicin (Sigma-Aldrich, St. Louis, MO, USA), were used as standard antibiotics for the investigation. Analytical grade piperine and piperlongumine standards (≥97.0% purity) purchased from TCI Europe N.V. (Zwijndrecht, Belgium) were used as standard compounds.

#### 4.4.2. Agar Disk Diffusion Method

Gram-positive and Gram-negative bacteria representing potential human pathogenic bacteria were used to investigate the in vitro growth inhibitory effects of *P. guineense* extracts. A total of twelve fractions from the fruit and leaf extracts of this plant were tested against eight bacterial strains. An agar disk diffusion method was applied for the initial screening [46]. The freeze dried extracts were re-dissolved in those solvents used originally for their extraction according to the method of Anyanwu and Nwosu [45]. Each of the extracts were prepared to a final concentration of 50 mg/mL (stock solution). Tetracycline and rifampicin were used as positive controls for the bacterial strains. The antibiotics were dissolved in methanol to a final concentration of 10 mg/mL used for the test. Sterile petri dishes (∅ =1 4 cm, VWR Finland) were used for the screenings. For the antibacterial screening, twenty-five mL of sterile base agar (Antibiotic agar No. 2, Difco, VWR Finland) was applied as a bottom layer into the sterile petri dishes using a sterile, serological pipet (Falcon, Becton Labware Europe) and allowed to solidify. Thereafter twenty-five mL of isosensitest agar (OXOID, Thermo Fisher Scientific) was applied as the top layer. The petri dishes were all allowed to solidify and then stored at +4 °C. The screening started with inoculation of the bacteria onto solid nutrient agar slants which were incubated overnight at +37 °C. The viable bacterial cultures from the agar slants were used to prepare an inoculum for the test. Bacteria from the agar slants were transferred into 2 mL of 0.9% (*w*/*v*) sodium chloride (NaCl) solution in a sterile glass tube using a sterile inoculation loop. 1 mL of the suspension was transferred into another sterile glass tube and the absorbance was measured at 625 nm (UV–Visible Spectrophotometer, Pharmacia LKB-Biochrom 4060). The other 1 mL of the suspension (sterile part) was diluted with the 0.9% NaCl solution so that the absorbance at λ = 625 nm becomes 0.1 (this suspension contains approximately 1.5 × 10^8^ CFU/mL). 200 µL of this diluted bacterial suspension was spread evenly on each petri dish and left to dry for some seconds with the lid open. A sterile cork borer (11 mm in diameter) was used to make six holes equidistantly from each other on the agar surface of the petri dishes. 200 µL of the 50 mg/mL plant extracts and 200 µL of the 10 m/mL antibiotics were carefully pipette into the holes respectively. Methanol, ethanol, hexane and chloroform, 200 µL of each, were used as solvent controls respectively. The petri dishes were transferred to the cold room and incubated at +4 °C for 1 h, thereafter they were incubated overnight at +37 °C. The diameters of the zones of inhibition were measured with a calliper under a petri dish magnifier and expressed as the mean of the diameters of three replicates ± SEM. 

The Activity index (AI) of the various extracts was measured in relation to the standard antibiotic tetracycline according to Fyhrquist et al. [48].

Thus, AI = Inhibition zone of the plant extract/Inhibition zone of standard antibiotic

#### 4.4.3. Microdilution Method for MIC and MBC Estimation 

From the result obtained from the agar disk diffusion assay, minimum inhibitory concentration (MIC) was estimated for some selected extracts based on their good antibacterial activity. MIC is considered to be the lowest concentration of an extract or compound resulting in the inhibition of at least 90% of the growth of a bacterial strain. MIC values were determined using a microdilution turbidimetric broth method based on the guidelines of Clinical and Laboratory Standards Institute [49]. Only extracts which expressed remarkable antibacterial activity in the agar plate method were tested for MIC. For the MIC evaluation, two-fold serial dilutions of the extracts from 2500–9.75 µg/mL were prepared in sterile Mueller-Hinton broth. Commercial pure compounds, piperine (1 mg/mL concentration in methanol) and piperlongumine (1 mg/mL concentration in methanol) were also two-fold serially diluted in Mueller -Hinton broth. Tetracycline and rifampicin were each two-fold serially diluted in Mueller-Hinton broth from 125–0.48 µg/mL respectively. 96 well microtiter plates (Nunc, Nunclone, Denmark) were used for the tests. The bacterial cultures were inoculated on Mueller-Hinton agar slants or in 5 mL Mueller-Hinton broth and grown for 24 h at +37 °C before the test. The turbidity of 1 mL of the overnight bacterial culture was measured at 625 nm using a UV–Visible Spectrophotometer type 1510 (Thermo Fisher Scientific Oy). The absorbance was adjusted to 0.1 at 625 nm (approximately 1.0 × 10^8^ CFU/mL). 100µL of this suspension A_625_ = 0.1 was further diluted 100-fold to get a working suspension or inoculum containing approximately 1.0 × 10^6^ CFU/mL. 100 µL of this inoculum and 100 µL of the plant extracts, pure compounds, antibiotics and solvent controls, were introduced into the 96 well microtiter plates. Therefore, each well contained 5 × 10^5^ CFU/mL. The solvent controls contained a maximum of 5% (*v*/*v*) of each solvent to be tested for toxicity. The growth control (GC wells) contained only the bacterial suspension and the test wells (T wells) contained plant extracts or pure compounds + bacterial suspension. Moreover, negative control wells were prepared for each plant extract/compound to be tested and these wells contained plant extract/pure compound and the broth only in order to subtract the light absorbance of extracts and compounds from the wells containing the corresponding extracts/compounds with bacteria. The microwell plates were incubated for 24 h in an incubator coupled to a shaker at +37 °C. The turbidity of the wells at 620 nm was recorded using a Victor 1420 spectrophotometer (Wallac, Finland). The MIC values were considered as the lowest concentration of each tested extract/compound or antibiotic which resulted in a 90% or more inhibition of the growth control. The tests were done in triplicate and the % growth was expressed as the mean of these triplicates ± standard error of mean (SEM). The minimum bactericidal concentration (MBC) was evaluated by pipetting 100 µL from those wells of the microtiter plate, which contained 2 and 4 times higher concentrations than the MIC values on petri dishes (∅ = 9 cm) containing Mueller-Hinton agar, where after, the dishes were incubated for 24 h at +37 °C. The MBC was taken as the lowest concentration where no visible growth on the petri dish was observed after the incubation.

## 5. Conclusions

The present study demonstrated that extracts of *P. guineense*, enriched in alkaloids, have potent antibacterial activity against a panel of Gram-positive and Gram-negative bacteria, including significant human pathogens. It has been established through this study that alkaloids are the main antibacterial compounds present in *P. guineense* leaves and fruits. However, water extracts and decoctions, which are frequently used as traditional medicinal preparations, were devoid of these alkaloids even at the highest tested concentration of 50 mg/mL and thus inactive against the studied bacteria. Our results revealed that *P. guineense* alcohol, chloroform and hexane extracts from the leaves and fruits are richsources of piperamide compounds which could help to combat bacterial infections. Thus our results encourage especially the use of alcohol extracts of *P. guineense* leaves and fruits in traditional medicine.

Our results warrant further in depth research on the antibacterial mechanisms of action of piperamide compounds, since this is not thoroughly studied. Plant derived compounds with new mechanisms of action could be used for the treatment of multi-drug resistant bacteria and *P. guineense* as well as other species of *Piper* could be sources for some of these compounds. Moreover, the alkaloids in *P. guineense* could be investigated for their anti-biofilm activities, including quorum sensing. The inhibitory activity observed with the various fractions and extracts against the tested bacteria in this investigation, warrants further exploration of the bioactive compounds from *P. guineense* as molecular scaffolds for new therapeutic agents in modern antimicrobial therapy, as well as on their potential as adjuvants together with conventional antibiotics to restore or to increase the effects of the antibiotics in current use. The possibility to use piperamide alkaloids from *P. guineense* (and other *Piper* spp.) for the treatment of drug resistant strains of *Pseudomonas aeruginosa*, *Staphylococcus aureus*, *Bacillus cereus* and *Salmonella enterica* should be explored.

## Figures and Tables

**Table 1:** Antibacterial effects of extracts of *P. guineense*, piperine and piperlongumine against Gram-negative bacteria. Results obtained with the agar diffusion method. Activity index (AI) describes the activity of the extracts and compounds in relation to tetracycline.

Extracts/Antibiotics	*E. aerogenes*	AI Tet.	*E. coli*	AI Tet.	*P. aeruginosa*	AI Tet.	*S. enterica*	AI Tet.	*P. mirabilis*	AI Tet.
PSMeOH	49.7 ± 0.33	1.10	20.7 ± 0.33	0.42	16.3 ±0.33	0.29	13.3 ± 0.67	0.31	13.7± 0.72	0.34
PSCHCL_3_	11.3 ± 0.33	0.25	20.3 ± 0.33	0.41	17.7 ±0.33	0.32	18.0 ± 0.0	0.32	11.3 ± 0.33	0.28
PSHex	34.7 ± 0.33	0.76	NA	NA	NA	NA	12.7 ± 0.71	0.23	11.7 ± 0.33	0.29
PSH_2_O *	NA	NA	NA	NA	NA	NA	NA	NA	NA	NA
PSHH_2_O	NA	NA	NA	NA	NA	NA	NA	NA	NA	NA
PSEthanol	30.2 ± 0.17	0.67	19.8 ± 0.24	0.40	NT	NT	21.7 ± 0.33	0.39	16.3 ± 0.32	0.40
PLMeOH	29.3 ± 0.33	0.65	11.3 ± 0.33	0.23	15.3 ± 0.33	0.28	12.0 ± 0.00	0.22	11.3 ± 0.31	0.28
PLCHCL_3_	11.3 ± 0.33	0.25	20.3 ± 0.33	0.41	21.3 ± 0.33	0.38	11.7 ± 0.33	0.21	10.7 ± 0.23	0.26
PLHex	17.8 ± 0.17	0.39	NA	NA	NA	NA	NA	NA	11.7 ± 0.33	0.29
PLH_2_O *	NA	NA	NA	NA	NA	NA	NA	NA	NA	NA
PLHH_2_O	NA	NA	NA	NA	NA	NA	NA	NA	NA	NA
PLEthanol	25.0 ± 0.00	0.55	16.3 ± 0.33	0.33	NT	NT	16.2 ± 0.24	0.29	13.8 ± 0.2	0.34
Piperine	18.7 ± 0.67	0.41	22.3 ± 0.32	0.45	15.7 ± 0.73	0.28	14.7 ± 0.32	0.26	16.0 ± 0.63	0.39
Piperlongumine	12.7 ± 0.65	0.28	20.0 ± 0.33	0.40	13.3 ± 0.32	0.24	11.3 ± 0.33	0.20	NA	NA
Rifampicin	38.0 ± 0.00	0.84	40.3 ± 0.34	0.81	45.3 ± 0.33	0.81	39.3 ± 0.33	0.71	34.3 ± 0.33	0.8
Tetracycline	45.3 ± 0.67	1.00	49.7 ± 0.33	1.00	55.7 ± 0.33	1.00	55.7 ± 0.33	1.00	40.7 ± 0.74	1.00

The diameter of the zones of inhibition in mm calculated as the mean of triplicates (n = 3) ± SEM (standard error of mean). PSMeOH, *P. guineense* fruit methanol extract; PSCHCL_3_, *P. guineense* fruit chloroform extract; PSHex, *P. guineense* fruit hexane extract; PSH₂O *, *P. guineense* fruit cold water maceration; PSHH₂O, *P. guineense* fruit hot water decoction; PSEthanol, *P. guineense* fruit ethanol extract; PLMeOH, *P. guineense* leaf methanol extract; PLCHCL_3_, *P. guineense* leaf chloroform extract; PLHex, *P. guineense* leaf hexane extract; PLH₂O *, *P. guineense* leaf cold water maceration; PLHH₂O, *P. guineense* leaf hot water decoction; PLEthanol, *P. guineense* leaf ethanol extract; AI Tet., activity index in relation to tetracycline; NA, not active and NT, not tested.

**Table 2:** Antibacterial effects of extracts of *Piper guineense*, piperine and piperlongumine against Gram-positive bacteria. Results obtained with the agar diffusion method. Activity index (AI) describes the activity of the extracts and compounds in relation to tetracycline.

Extracts/Antibiotics	*Sarcina* sp.	AI Tet.	*S. aureus*	AI Tet.	*B. cereus*	AI Tet.
PSMeOH	29.83 ± 0.17	0.72	15.7 ± 0.33	0.32	23.7 ± 0.32	0.44
PSCHCL_3_	31.67 ± 0.33	0.76	17.3 ± 0.67	0.35	15.7 ± 0.33	0.29
PSHex	37.67 ± 0.33	0.90	17.3 ± 0.33	0.35	19.7 ± 0.33	0.36
PSH_2_O *	10.67 ± 0.33	0.26	NA	NA	NA	NA
PSHH_2_O	17.67 ± 0.33	0.42	NA	NA	NA	NA
PSEthanol	28.33 ± 0.33	0.68	22.67 ± 0.33	0.46	24.5 ± 0.33	0.45
PLMeOH	26.17 ± 0.17	0.63	14.7 ± 0.33	0.30	29.3 ± 0.33	0.54
PLCHCL_3_	25.67 ± 0.33	0.62	16.7 ± 0.67	0.34	15.3 ± 0.32	0.28
PLHex	33.67 ± 0.33	0.81	15.0 ± 0.00	0.30	24.7 ± 0.31	0.45
PLH_2_O *	10.67 ± 0.33	0.26	NA	NA	NA	NA
PLHH_2_O	14.33 ± 0.33	0.34	NA	NA	NA	NA
PLEthanol	25.17 ± 0.17	0.60	17.5 ± 0.29	0.35	28.2 ± 0.24	0.52
Piperine	27.67 ± 0.33	0.66	18.3 ± 0.33	0.37	12.7 ± 0.33	0.23
Piperlongumine	23.67 ± 0.33	0.57	15.3 ± 0.33	0.31	15.7 ± 0.3	0.29
Rifampicin	39.33 ± 0.33	0.94	48.3 ± 0.33	0.98	50.7 ± 0.33	0.93
Tetracycline	41.67 ± 0.33	1.00	49.3 ± 0.33	1.00	54.3 ± 0.32	1.00

The diameter of the zones of inhibition in mm calculated as the mean of triplicates (n = 3) ± SEM (standard error of mean). For the key to other abbreviations, see Table 1. PSH₂O *, *P. guineense* fruit cold water maceration and PLH₂O *, *P. guineense* leaf cold water maceration.

**Table 3:** Minimum inhibitory concentration and minimum bactericidal concentrations (MIC and MBC) in µg/mL of fruit and leaf extracts of *Piper guineense* and the alkaloids piperine and piperlongumine.

Plant Extracts and Antibiotics	*Sarcina* sp.	*S. aureus*	*B. cereus*	*P. mirabilis*	*E. aerogenes*	*E. coli*	*P. aeruginosa*	*S. enterica*
PSMeOH								
MIC	**39**	625	**78**	1250	**39**	1250	1250	625
MBC	78	1250	156	2500	78	2500	2500	1250
PSCHCL_3_								
MIC	156	156	625	2500	312	312	**78**	**78**
MBC	312	312	1250	NT	625	625	156	156
PSHex								
MIC	**39**	**78**	625	2500	**39**	NA	NA	2500
MBC	78	156	1250	NT	78	NA	NA	NT
PSEthanol								
MIC	**39**	**78**	**39**	**78**	**78**	156	NT	**78**
MBC	78	156	78	156	156	312	NT	156
PLMeOH								
MIC	**39**	312	**78**	1250	**39**	1250	1250	625
MBC	78	625	156	2500	78	2500	2500	1250
PLCHC_3_								
MIC	**78**	625	625	2500	312	312	**78**	**78**
MBC	156	1250	1250	NT	625	625	156	156
PLHex								
MIC	**19**	**78**	625	1250	**78**	NA	NA	NT
MBC	39	156	1250	2500	156	NA	NA	NT
PLEthanol								
MIC	**39**	**78**	**39**	**78**	**78**	312	NT	**78**
MBC	78	156	78	156	156	625	NT	156
Piperine								
MIC	**19**	**39**	625	**78**	**39**	**19**	156	**78**
MBC	39	78	1250	156	78	39	312	156
Piperlongumine								
MIC	**9.7**	156	**78**	NA	156	**39**	312	625
MBC	19	312	156	NA	312	78	625	1250
Tetracycline								
MIC	****0.48****	**0.97**	**0.48**	**1.95**	**0.48**	**0.48**	**0.48**	**0.97**
Rifampicin								
MIC	**0.97**	**0.97**	**0.48**	**0.97**	**0.48**	**0.48**	**0.48**	**0.48**

For the key to other abbreviations, see Table 1. The extracts with marked MIC values are in bold.

**Table 4:** HPLC-DAD and UHPLC/QTOF-MS data of alkaloids from a *Piper guineense* leaf hexane extract.

*Piper guineense* Peak Number and Name of Compound	Molecular Formula	Rt HPLC-DAD (min)	Rt UHPLC (min)	Measured Mass	Calculated Mass	[M + 1]^+^	Other Ions *m*/*z*	UVλ Absorption Max.	Accuracy (ppm)
**1**. Unknown		11,840	2842	187,0306 *		165,0487		214, 236	-
**2**. Unknown (Ellagitannin?)		16,383	6173	312,1211 *		290,1392		210, 270	-
**3**. Unknown		23,461	6373	390,1516 *		368,1697			-
Unknown		33,389	12,567	231,0993 *		209,1174		220, 238, 320, 355	-
**6**. Unknown		36,456	13,267	187,0277		165,0458	143,068; 111,0420		-
**7**. Dihydropiperylin or piperlonguminine	C_16_H_19_NO_3_	36,852	12,967	296,1250 *	273.1365	274.1431Q	2(M)^+^-23: 569,2628	210, 235, 284	−4,2212
**7.1**. Unknown			13,267	187,0277		165,0458	143,068; 111,0420		-
**8**. Piperylin	C_16_H_17_NO_3_	38,563	13,567	294,1100 *	271,1208	272,1281Q	2(M)^+^-23: 565,2321	210, 240, 308, 344	−2,0740
**9**. Piperidine derivative		39,226	13,783	326,1374 *		304,1555	2(M)^+^23:629,2844	210, 234, 300	
**10**. Dihydropiperlonguminine	C_16_H_21_NO_3_	41.5	14,066	298,1337 *	298,1419	276,1518Q		210, 235, 300	27,50
**10b**. Piperine derivative		39,249	14,432	324,1211 *		302,1392	2(M)^+^-23: 625,2522	210, 235, 288	
**11**. Unknown piperamide alkaloid		40,447	15,049	358,1528 *		336,1709	143,0682	210, 220, 246, 276, 355	-
**11b**. Unknown piperamide alkaloid		40,447	14,832	317,1330 *		295,1511	143,0644	210, 220, 246, 276, 355	-
**12**. Unknown			16,031	247,1612		225,1793			-
**13**. 4,5-dihydropiperine	C_17_H_21_NO_3_	42,215	15,515	310,1417 *	287,1521	288,1598Q		210, 235, 287	−0,6771
**15**. Piperine	C_17_H_19_NO_3_	44,809	16,647	308,1245 *	285,1365	286,1426Q		210, 240, 310, 342	−5,7119
**16**. Dihydrowisanine	C_18_H_23_NO_4_	45,234	16,847	340,1530 *	317,1627	318,1711Q		210, 236, 306, 340	1,5581
**17**. Unknown			17,197	247,1659		225,1840			-
**18**. Wisanine	C_18_H_21_NO_4_	48,433	17,846	338,1371 *	315,1471	316,1552Q	247,1662	218, 253, 304, 370, 372	0,8281
**19**. Unknown			18,795	243,1299 *		221,1480			

UHPLC/QTOF-MS in positive ion mode was used, * = M + 23, Q = M + 1, ppm = mass accuracy in parts per million.All the results with an asterisk are [M + 23]^+^ results, they are adducts with sodium (mass = 23 for sodium). Ions for compounds 5 and 14 in HPLC-DAD, Figure 1,were not detected in our UHPLC/QTOF MS system and thus these compounds are left away from Table 4.
